# Polo, Greatwall, and Protein Phosphatase PP2A Jostle for Pole Position

**DOI:** 10.1371/journal.pgen.1002213

**Published:** 2011-08-11

**Authors:** Elvan Boke, Iain M. Hagan

**Affiliations:** CRUK Cell Division Group, Paterson Institute for Cancer Research, Manchester, United Kingdom; Stowers Institute for Medical Research, United States of America

Commitment to mitosis is driven by activation of the Cdk1-Cyclin B protein kinase complex known as Mitosis Promoting Factor (MPF). MPF activation promotes downstream protein kinases that control the formation and function of the mitotic spindle. These kinases include members of the NIMA, Greatwall/Scant, Polo, and Aurora kinase families. Each kinase often phosphorylates multiple targets. Sophisticated dependency relationships enable a single kinase to promote distinct events at the same or successive stages of mitosis. Understanding mitosis means cataloguing each target for each kinase and deciphering the interplay between the ensuing pathways. Two papers in this issue of *PLoS Genetics* show that the ability of Greatwall/Scant kinase to generate an inhibitor of Protein Phosphatase 2A (PP2A) underpins an antagonistic interplay between Greatwall and Polo in *Drosophila* [1], [2].

## Polo Kinase

The mitotic kinases Polo, Aurora, and Greatwall were identified through *Drosophila* genetics. “Polo” describes the circular profile of chromosomes associated with the monopolar spindles in *polo* mutants [3]. Humans have four Polo kinases. *Drosophila* Polo is considered to be analogous to mammalian Plk1 [4]. Plk1 participates in a multitude of functions ranging from MPF activation, through cohesin destruction at the metaphase-anaphase transition, to the timing and execution of cytokinesis. The defining feature of a Polo kinase is a Polo Box Domain (PBD) that docks Polo kinase to target proteins. In the majority of cases, Plk1's PBD binds to a phosphorylated motif in which the phosphorylation site matches the MPF consensus sequence. Thus, Polo must usually wait for targets to be phosphorylated by MPF before it can impose its authority [4].

## Greatwall Kinase

*greatwall* mutants fail to correctly condense their chromosomes, leading to the naming of the kinase Greatwall as a protector of chromosome integrity [5]. While most closely related to NDR kinases, the presence of a large loop between kinase domains VII and VIII is a defining feature of Greatwall kinases [5]. Studies in cell-free *Xenopus* egg extracts demonstrate that Greatwall activity is critical to drive mitotic commitment [6]. Greatwall inhibits the PP2A-B55δ protein phosphatase complex [7], [8]. PP2A-B55δ activity oscillates as cells transit the cell cycle. It is high in interphase and low in mitosis [9], [10]. The activity of PP2A-B55δ counteracts MPF's efforts to promote and maintain the mitotic state [7], [8], [10]. PP2A-B55δ must therefore be switched off before a stable mitotic state can be achieved, making PP2A-B55δ inactivation an integral part of mitotic commitment [7], [8], [10], [11]. Once mitosis is complete, PP2A re-activation dephosphorylates MPF targets to drive mitotic exit [7], [8], [10]. Recent studies established that phosphorylation of the related molecules Endosulfine and Arpp19 by *Xenopus* Greatwall converts them into potent PP2A-B55δ inhibitors [12], [13]. Consequently, Greatwall activation upon mitotic commitment effectively locks the cell into the mitotic state.

Two further functions have been ascribed to Greatwall kinases: the modulation of RNA stability during G0 in budding yeast and, as discussed below, the antagonism of Polo kinase activity in *Drosophila* [14], [15]. Rim15, the budding yeast Greatwall kinase, phosphorylates yeast Endosulfine (and human Ensa and Arpp19) at the equivalent site to the *Xenopus* kinase, and yeast phospho-Endos subsequently binds components of a ribosome-associated protein complex to control mRNA stability [15]. Thus, it is plausible that phospho-Endos/Arrp19 may yet be found to target molecules other than PP2A in cell cycle control in other systems. The reduction in the protein levels of both Polo and the meiotic Cdc25 homologue Twine in *Drosophila endosulfine (endos)* mutants is consistent with altered translation in this system [16].

## *Drosophila* Greatwall and Polo an Uneasy Pairing

The antagonistic relationship between Polo and Greatwall was revealed by a second-site mutant (*Scant*) that failed to complement the *polo^1^* mutant with respect to embryonic viability [14]. *Scant* is a dominant, hyper-activating allele of *gwl* (denoted *gwl^scant^*) [17]. One component of this synthetic lethality may lie in the failed association of one centrosome with the prophase spindles in *polo^1^*/+ *gwl^scant^*/+ embryos. Increasing the *polo:gwl^scant^* ratio by duplication of *polo* ^+^ suppressed this phenotype, while reducing this ratio by using a Polo inhibitor enhanced it [14], [17], [18]. Moreover, *polo* ^+^ duplications restore fertility to *polo^1^*/+ *gwl^scant^*/+ females [17]. Taken together, these data demonstrate that the phenotype of the *gwl^scant^* mutant can be modulated by altering the dose of *polo^+^*.

## Conservation of the Greatwall Control of PP2A Activity by Endos Phosphorylation

Rangone et al. demonstrate the ability of *Drosphila endos* mutants to phenocopy *Scant* intragenic supressors [1], [14]. They show the in vitro phosphorylation of *Drosophila* Endos by *Drosophila* Greatwall, supporting the view that *polo^1^*/+ *gwl^scant^*/+ embryos die because *gwl^scant^*generates excessive levels of phospho-Endos, which subsequently block PP2A activity. A reduction in the level of PP2A regulatory or catalytic subunits also reduces the fertility of *polo^1^*/+ *gwl^scant^*/+ females. The *Drosophila* and *Xenopus* stories mirror each other in two important respects. Of the four PP2A regulatory subunits, it is only the complex harbouring the B55 subunit (encoded by *twins*) that participates in Endos control [1]. Second, the introduction of *Scant* into *Xenopus* Greatwall increases its interphase activity and promotes premature commitment to mitosis [19].

Wang et al. derived similar conclusions from a very different starting point by systematically seeking deficiencies that enhanced the fertility defect of *polo*-compromised and *Scant* flies [2]. After realising that the strongest of the six hits they obtained corresponded to a *twins* deletion, they employed a genetic analysis to demonstrate that the Greatwall/Endos/PP2A relationship is conserved in *Drosophila* [2].

These two independent screens used opposite approaches (enhancers versus suppressors) to study Polo and Greatwall and found opposing components of the same regulatory network. The search for suppressors of *gwl^scant^* identified mutations in *endos*, i.e., mutations that increased PP2A activity. In contrast, the search for enhancers of the *Scant* phenotype identified PP2A and *twins* mutations that reduced it. More broadly speaking, the biochemical dissection of *Xenopus* extracts and genetic dissection of *Drosophila* come to remarkably consistent conclusions: the Greatwall/Endos/PP2A switch. The meiotic progression defects of *twins* and *endos* mutants and *greatwall* over-expressors suggest that these parallels extend to the control of the maintenance of the meiotic state in *Drosophila* [2], [16].

## In Pursuit of Poles

The enhancement of the fertility defect of *gwl^scant^* by the same deletions that enhance *polo* in the Wang et al. study [2] both provides firm affirmation of the antagonism between Greatwall and Polo and poses the question “Why are *polo* mutants so sensitive to a reduction in PP2A levels?” The answer may be linked to the centrosome retention phenotype. Defective centrosome attachment is a common occurrence during the syncitial divisions of mitotic mutants, suggesting that it may simply be indicative of compromised spindle function (D. M. Glover, personal communication). However, the role this phenotype played in unlocking the Polo Greatwall/Endos/PP2A relationship suggests that the possibility of a functional link merits consideration.

## Nuclear Envelope Integrity during Syncitial Divisions

**Figure 1:**
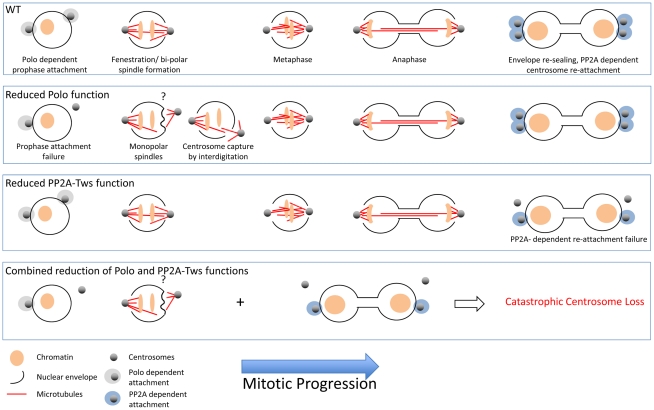
In wild-type syncitial embryos, the migration of centrosomes along the nuclear surface before their activation to nucleate microtubules co-incides with localised fenestration of the nuclear envelope beneath them. This pore remains open until completion of karyokinesis, whereupon the envelope re-seals and the centrosomes re-attach. Thus, the microtubules emanating from each centrosome are able to interdigitate and drive anaphase elongation, culminating in karyokinesis. As indicated in the appropriate panels, the association of the centrosomes fails during prophase in *polo* mutants and between anaphase and karyokinesis in PP2A-related mutants. The attachment is represented in the cartoons as a sphere around the centrosome to reflect our ignorance of the nature of the attachment regulated by these enzymes at each stage. The defect could be an inability to physically generate/recruit anchors on the centrosomes or an inability to activate anchors at the nuclear surface. For example, it is known that dynein is required for centrosome anchorage. Alternatively, the anchors may be activated at the centrosome and transported to the envelope along microtubules. The question mark above the monopolar spindle cartoon in the Polo reduction box reflects the uncertainty of the detail of the phenotype: fenestration or attachment? It is currently unclear whether the defect at this stage is truly an attachment defect. It could arise from a problem in generating the holes in the envelope to enable the microtubules emanating from the centrosome to meet one another and form the spindle. If the microtubules can not penetrate the envelope to establish a bipolar spindle, the force arising from polymerising microtubules repeatedly pushing against the intact envelope would push the centrosome away. This contrasts with the normal situation where the microtubule-driven forces arising from bipolar spindle formation would pull the two poles towards one another and keep the centrosomes attached. If fenestration is defective, then the “attachment” defect is rather a *polo*-directed fenestration deficiency. If so, then it is just the PP2A-related regulated events that represent a true attachment defect.

The nuclear envelope remains largely intact throughout syncitial divisions of the early embryo. Limited fenestration at the spindle poles, beginning at pro-metaphase, enables the microtubules emanating from the centrosomes to capture kinetochores and form the central spindle [20]. These pores close following spindle disassembly. Newly duplicated prophase centrosomes migrate away from one another on the surface of the nuclear envelope to straddle the prophase nucleus just before fenestration grants them access to the nucleoplasm (Figure 1, WT).

## Centrosome Detachment in *polo* Mutants

Reducing Polo activity can cause one of the two centrosomes to disassociate from the nuclear envelope around the time of fenestration [1], [2], [17], [18]. The pushing forces generated by microtubules emanating from this detached centrosome distort the nuclear envelope [2] (Figure 1, Polo deficiency). As similar distortions occur immediately before fenestration in wild-type embryos, the centrosome detachment phenotype may reflect a requirement for Polo to drive fenestration [20]. Alternatively, the problems in centrosome retention may lie in Polo's well-characterised role in promoting centrosome maturation [4]. The centrioles in the two centrosomes at either spindle pole are not of equivalent ages, making it possible that one is insufficiently mature to retain its grip on the nucleus when Polo activity is decreased. In this scenario, insufficient Polo leads to an inability to either recruit or activate anchors at either the centrosome or the nuclear envelope, or promote localised fenestration.

## Cumulative Action of Greatwall and Polo

So how does the Greatwall/Endos/PP2A pathway impact centrosome retention? The name of the dominant Greatwall mutation, *Scant* (“Scott of the Antarctic”), holds the key: *polo^1^*/+ *gwl^scant^*/+ mutants have greater problems in retaining the association between the centrosome and the nuclear envelope (i.e., in finding the poles) than do *polo^1^*/+ +/+ mutants. *Scant* was named after British explorer Captain Scott, who set off on an unsuccessful mission to the South Pole.

As *gwl^scant^* is a hyperactive mutation, the enhancement of the detachment phenotype of *polo* mutants might indicate that PP2A assists Polo in promoting prophase attachment. However, this appears not to be the case, as PP2A mutants have no defect in prophase attachment [2]. Rather, PP2A single mutants display attachment defects at a later stage of mitosis; from late anaphase [2] (Figure 1, PP2A-Twins deficiency). In other words, PP2A-mediated dephosphorylation promotes centrosome docking to the envelope during mitotic exit. This timing is consistent with the distribution of Greatwall in the immediate vicinity of the nucleus throughout anaphase before nuclear import upon spindle dissolution [17]. If phospho-Endos is a short lived entity, removal of Greatwall from the vicinity of the envelope would generate a local burst of PP2A activity in the region where the centrosome binds the envelope.

## Parallel or Sequential Pathways?

Are the two attachment phenotypes (Polo-driven association in prophase and PP2A-driven association during mitotic exit) connected (Figure 1, Polo/PP2A-Twins deficiency)? Wang et al. propose that they are not. Cells recover from the *polo*-dependent prophase centrosome loss by re-capturing the errant centrosome on their anaphase spindles [2]. The association of centrosomes with the envelopes of PP2A mutants before anaphase suggests that re-capture occurs in PP2A mutants as well [2]. However, they suggest that recovery becomes catastrophically challenging when detachment repeatedly occurs at distinct stages of the cycle in double mutants of *polo* and PP2A.

Alternatively, Rangone et al. provide plausible arguments for a functional link between the Polo and PP2A-driven dephosphorylation to promote centrosome attachment after mitotic exit. There are precedents in which Polo recruitment and subsequent function is driven by dephosphorylation or the dephosphorylated state. The structural component of the anaphase mid-zone, PRC1, is unable to recruit Polo until a Cdk phosphorylation site is dephosphorylated in anaphase [21]. Similarly, MAP205 must be dephosphorylated to bind and sequester Polo in interphase [18]. MAP205 normally binds Polo throughout interphase to contribute to its inactivation by keeping it away from targets; however, the key issue in the context of rising PP2A activity during mitotic exit is the antagonism between Cdk phosphorylation and the recruitment of Polo to dephosphorylated substrates.

Clearly, greater insight into the molecular basis of centrosome attachment is required before we can resolve these possibilities.

## Perspectives

An overriding message from these studies is the power of *Drosophila* genetics to reveal the interplay between signalling networks. It is no accident that “cell cycle speak” has accumulated abstract names such as “Polo”, “Aurora”, “Scant”, and “Greatwall” at the heart of its everyday vocabulary. *Drosophila* genetics remains at the forefront of our attempts to piece together multiple regulator target relationships into a holistic view of the networks that constitute mitotic control. The focus and simplicity of the *Scant* screens in particular suggest that many insights into the Greatwall/Polo/PP2A axis will continue to emerge from this approach. In the immediate future, the attenuation of the phospho-Endos inhibitory signal is a particularly pressing objective for the field.
